# Structural and Material Optimization for Automatic Synthesis of Spine-Segment Mechanisms for Humanoid Robots with Custom Stiffness Profiles

**DOI:** 10.3390/ma12121982

**Published:** 2019-06-20

**Authors:** Adam Ciszkiewicz, Grzegorz Milewski

**Affiliations:** Institute of Applied Mechanics, Cracow University of Technology, 31-155 Kraków, Poland; milewski@mech.pk.edu.pl

**Keywords:** structure optimization, parameters estimation, material optimization, intervertebral joint

## Abstract

Typical artificial joints for humanoid robots use actual human body joints only as an inspiration. The load responses of these structures rarely match those of the corresponding joints, which is important when applying the robots in environments tailored to humans. In this study, we proposed a novel, automated method for designing substitutes for a human intervertebral joint. The substitutes were considered as two platforms, connected by a set of flexible links. Their structural and material parameters were obtained through optimization with a structured Genetic Algorithm, based on the reference angular stiffnesses. The proposed approach was tested in three numerical scenarios. In the first test, a mechanism with angular stiffnesses corresponded to the actual L4–L5 intervertebral joint. Scenarios 2 and 3 featured mechanisms with geometry and structure comparable to the joint, but with custom stiffness profiles. The obtained results proved the effectiveness of the proposed method. It could be employed in the design of artificial joints for humanoid robots and orthotic structures for the human spine. As the approach is general, it could also be extended to different body joints.

## 1. Introduction

**Figure 1:**
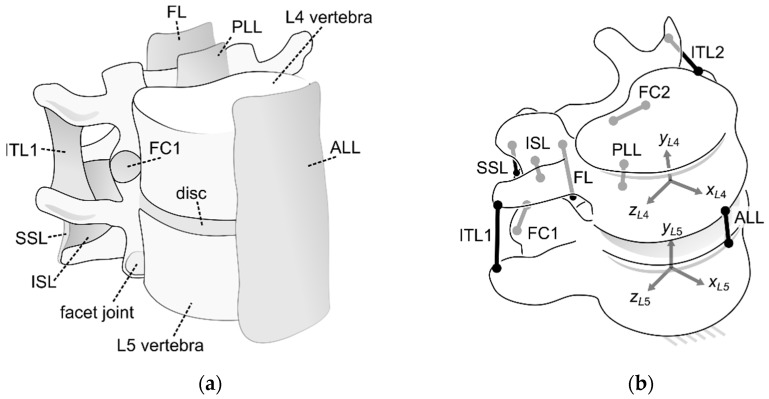
(**a**) The L4–L5 intervertebral joint (IJ); (**b**) the ligament system of the IJ (ligaments substituted with cables).

The spine is one of the most important structures in the human body. It serves as a column supporting the organs and as a central hub for other skeletal systems of the body. It is formed by 33 vertebrae, which can be divided into intervertebral joints (IJ) (see Figure 1a). These joints are composed of a set of ligaments, a disc, and two vertebrae [1,2]. Nine major ligaments can be distinguished in IJ: The anterior longitudinal ligament (ALL), posterior longitudinal ligament (PLL), supraspinous ligament (SSL), interspinous ligament (ISL), flaval ligament (FL), two intertransverse ligaments (ITL1, ITL2), and two capsular ligaments (FC1, FC2) [3]. Furthermore, two bony structures are located on the posterior side of each vertebra. They are called facet joints and mostly constrain the relative axial rotation of the vertebra. The ligaments resemble nonlinear cables in their behavior. The disc is a viscoelastic structure, which transfers mostly compressive loads in the IJ.

With the advent of humanoid robotics, an increase in research studies regarding artificial joints for humanoids can be observed. Since these robots are made to function in environments typically tailored for humans, the behavior of their joints is of vital importance. Usually, the closer the artificial joint is to its real counterpart, the better. Nevertheless, considering how complex the actual joints are, this can be difficult to achieve. In the following section, some of the more popular approaches for artificial joints for the spine or its segments are presented. Furthermore, the section also contains a description of the available models for the spine and its segments as they may serve as an inspiration for artificial joints.

### Literature Review

The importance and complex nature of the spine and its joints have translated to many models of it in literature. These models can be divided into two groups: Finite element models (FEM) and multibody models (MBS). The FEM provide very accurate results, useful when assessing the stress-strain state of the joint [3,4], and can also be used to design artificial discs [5,6,7]. Nevertheless, due to high computational load, the FEM is usually applied to structures with limited mobility. On the other hand, the MBS is well suited for systems experiencing large displacements, such as a lumbar spine with muscle system [8,9,10]. However, complex viscoelastic systems are difficult to analyze with this method. Therefore, the disc in the IJ has been described with a stiffness matrix [11,12] or with a set of flexible and/or damping elements [13,14].

As pointed out, both the human spine and its IJs are complex in their structure, featuring a plethora of elements, including nearly rigid bones, cable-like ligaments, and deformable, nonlinear structures. These elements give the IJs their very specific load responses and passive stability [15]. Nevertheless, their sheer number and complex behavior make them very difficult to substitute in technical solutions for humanoid robots. This problem is a very important one as the closer the artificial joints get to the original ones, the more human the response of the robot. In the literature, many solutions to this problem were presented for the spine. The simplest one has been to represent the spine, or its segments, with a series of 1 to 3 revolute joints or ball joints [16,17,18,19,20,21]. Other research groups have employed more complex parallel structures for this purpose [22,23,24]. Such parallel structures have also been applied in other body joints [25,26,27]. These kinematic approaches only constrain the motion of the mechanism. To give it a passive stiffness response to load, additional elements are necessary. In [16], the authors augmented the ball joint with a set of flexible springs and a rubber layer. This contrasts with the approach in [28,29]. In these studies, the proposed mechanisms have been based on a flexible central element—a beam or a spring. This element has been complemented by other rigid links and cables. The discussed structures have a stiffness response. Nevertheless, the spine served for them mostly as an inspiration. Its actual responses to load have not been employed in the design process. A different approach was presented in [30]. In this study, substitute mechanisms for the IJ were obtained based on the ligament system of the IJ through a two-step optimization with Genetic Algorithm. As the ligament system contained only cable elements, the initial search optimized the structure by changing some cables to springs and deactivating unnecessary ones. The optimized structure was then subjected to a second search, this time for optimal parameters. The method returned a 7-link mechanism with angular stiffnesses mimicking that of the actual IJ. Nevertheless, the separation of structure optimization and parameters estimation can be limiting, especially when considering mechanisms with custom load responses. 

The aim of this study was to extend and generalize the previously published methodology in [30] by applying structured Genetic Algorithm (sGA) [31] for simultaneous optimization of structure and its parameters of mechanisms with flexible links. The approach was then tested in three numerical scenarios, including mechanisms with custom load responses:

1. A mechanism with angular stiffnesses comparable to the L4–L5 IJ;
2. A mechanism with geometry comparable to the IJ, but increased stiffness in flexion;
3. A mechanism with geometry comparable to the IJ, but significantly reduced range of motion in lateral bending and axial rotation.

Our main contributions to the existing research were in:

- Successfully applying sGA to simultaneous optimization of structural and material parameters of flexible parallel structures for use in humanoid robots, based on actual human body joints;
- Obtaining custom mechanisms with geometry close to that of the IJ, but with significantly different responses to static loads.

## 2. Materials and Methods

The IJ of the human spine contains flexible elements (ligaments), viscoelastic structures (a disc), and facet joints (bony constraints). Due to the sheer number of elements, it would be difficult to reproduce them fully in an artificial joint for use in humanoid robots. Such a structure would be prone to failures. Therefore, our aim was to obtain a simple substitute, composed only from linear springs, cables, and two rigid platforms through optimization. This mechanism was to be capable of reproducing the angular stiffnesses of the IJ. To simplify the search and ensure that the output geometry was close to that of the reference, the bounds for the optimization were based on the ligament system, as presented in Figure 1b. The optimization procedure was allowed to deactivate selected ligaments and change some of them from cables to springs. The following paragraphs describe the methodology to solve parallel mechanisms with flexible links in statics, formulate an objective function used to rate the mechanisms, and employ sGA, a global optimizer for structure, geometry, and material parameters of the mechanism.

### 2.1. Solving Three-Dimensional, Elastostatic Problems

The problem of solving elastostatic problems numerically has been explored in detail in our previous work [30]. This section only briefly summarizes the previously published methodology. We assumed that the considered mechanisms were composed of two rigid platforms and multiple flexible links connecting them—linear springs and cables. Each platform had its own reference frame and the lower platform was stationary. As no constraints were applied on the moving platform, its position and location were defined by 6 geometric variables—three linear displacements forming the position vector ***p***:

(1) $$p={\left[\begin{array}{ccc} p_{x} & p_{y} & p_{z} \end{array}\right]}^{T},$$

and 3 angular displacements used to obtain the rotation matrix:

(2) $$R=\left[\begin{array}{ccc} c\alpha c\gamma +s\alpha s\beta s\gamma & -s\alpha c\gamma +c\alpha s\beta s\gamma & -c\beta s\gamma \\ s\alpha c\beta & c\alpha c\beta & s\beta \\ c\alpha s\gamma -s\alpha s\beta c\gamma & -s\alpha s\gamma -c\alpha s\beta c\gamma & c\beta c\gamma \end{array}\right],$$

where ***R*** = the rotation matrix from the moving to the stationary platform reference frame, sα = sinα, cα = cosα. The sequence was assumed after [32,33], while the angles α, β, γ corresponded to the flexion, the lateral bending, and the axial rotation of the moving platform. The forces and the moments acting on the moving platform and caused by linear springs were computed using the following equations:

(3) $$\begin{array}{ll} F_{s}=-k_{s}\Delta l_{s}, & F_{s}^{o}=\frac{b_{s}-a_{s}}{\left\|b_{s}-a_{s}\right\|},\\ F_{s}=F_{s}^{o}F_{s}, & M_{s}=b_{s}\times F_{s}, \end{array}$$

where ***F_s_*** (***M_s_***) = the force (moment) generated by the linear spring, *k_s_* = the stiffness parameter for the spring element, Δ*l_s_* = the change of the spring element length, and ***a_s_*** (***b_s_***) = the position vector of the spring element attachment to the lower (upper) platform.

The methodology for linear cables was analogous. The obtained loads were then inputted into the equilibrium equations for the moving platform:

(4) $$\left\{ \begin{array}{l} \sum_{i=1}^{n}F_{ci}+\sum_{j=1}^{m}F_{sj}+F_{ext}=0\\ \sum_{i=1}^{n}M_{ci}+\sum_{j=1}^{m}M_{sj}+M_{ext}=0 \end{array}\right.,$$

where ***F_s_*** (***M_s_***) = the forces (moments) generated by the linear springs, ***F_c_*** (***M_c_***) = the forces (the moments) generated by the linear cables, ***F_ext_*** (***M_ext_***) = the external force (moment) acting on the upper platform, and *n* (*m*) = the number of the springs (cables). 

The 6 variables defining the location of the moving platform were the unknown quantities in the equilibrium equation (Equation (4)). These equations were solved numerically using *fsolve* from Matlab under external moment loads of magnitudes up to 10 Nm, acting along all 3 axes of the stationary reference frame.

### 2.2. The Objective Function

In general, every optimization problem requires the decision variable vector ***x***, also referred to as a solution, and an objective function, which takes in the vector ***x*** and rates it. In this study, the vector ***x*** contained the full information required to describe a mechanism (see the next section). To rate the mechanisms, based on their static behavior, the following objective function was used in minimization:

(5) $$\underset{x}{\min}h(x)=w_{1}diff_{\alpha }+w_{2}diff_{\beta }+w_{3}diff_{\gamma }+w_{4}not\_passed,$$

where *h* = the objective function, ***w_i_*** = the weight *i* (here, ***w_i_*** = 1 (*i* = 1.3), *w*_4_ = 2), *not_passed* = the number of loads, for which the solver did not converge to a solution in 200 iterations, and:

(6) $$diff_{u}=\frac{\sum_{i=1}^{11}\left|\Delta u\left(M_{xi}\right)-\Delta u_{ref}\left(M_{xi}\right)\right|}{\max(\Delta u_{ref})-\min(\Delta u_{ref})},$$

where *u* ∈ {α = flexion, β = lateral bending, γ = axial rotation}, thus, *diff*_α_ = the flexion displacement indicator; Δα = the angular displacement obtained from the mechanism at flexion moment, *M_αi_* from 0 Nm to 10 Nm (Δ*M*_α*i*_ = 1 Nm), and Δα*_ref_* = the reference angular displacement measured on the actual joint or obtained from a verified joint model at flexion moment, *M*_α*i*_ from 0 Nm to 10 Nm (Δ*M*_αi_ = 1 Nm).

The objective function computed the difference between the angular displacements of the mechanism and the reference under the specified moment loads. Furthermore, it favored mechanisms, which presented no difficulties when solving for their elastostatic behavior. Low values of the objective corresponded to mechanisms with angular stiffnesses close to that of the reference IJ. Since multiple loading conditions were considered, the undertaken problem could be seen as a multi-objective one. In this study, we opted for one of the simpler strategies for multi-objective optimization, which was to sum up the objectives with appropriate weights, as seen in Equation (5). There were several reasons behind our choice. Firstly, this type of strategy—weighted summation—was successfully employed in many applications for the mechanism’s optimization [30,32,34,35,36,37]. Secondly, 3 out of 4 of the objectives considered in our study were of the same type. They represented the angular displacements in three planes, scaled by their typical ranges found in the IJ. The fourth objective allowed us to consider mechanisms that did not solve for all the loads with a penalty, instead of excluding them from the solution pool. These specimens had a small chance to reproduce during the optimization, which increased the diversity within the solutions.

### 2.3. Obtaining the Bounds for the Optimization Procedure

Before addressing the methodology from an algorithmic side, it was necessary to make initial assumptions regarding the mechanisms. As mentioned previously, the bounds for the optimization procedure were generated based on the actual ligament system of the L4–L5 IJ. In reality, the ligaments are geometrically complex and of nonlinear material response. Furthermore, they transfer mostly tensile loads. To use them as bounds for the procedure, it was necessary to assume simplified models. In the literature, many approaches to this problem can be found. In FEM modelling, the ligaments are substituted with multiple finite elements, often described with nonlinear constitutive laws. In the MBS models, the ligaments are usually replaced with cables with two point attachments. The behavior of these cables is defined not by a constitutive law, which relates stresses to strains, but by stiffness, which relates the forces acting on the ends of the cable to its elongation. Stiffness can be seen as a parameter, which encapsulates selected aspects of the geometry of the ligament (cross-section, free length) and its material properties, such as Young’s modulus for linear materials. 

**Figure 2:**
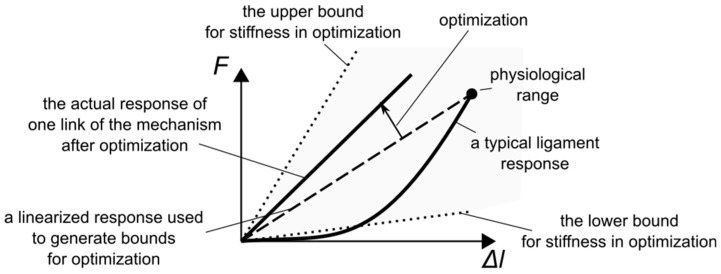
The force-elongation curves for an actual ligament, a linearized cable used in generating bounds for the optimization procedure, and a cable after optimization.

The force-elongation curve, used to obtain the stiffness, for a typical ligament reveals a nonlinear load response [38,39], as seen in Figure 2. This nonlinearity can be captured by nonlinear cable models for the ligaments. Nevertheless, in this study, the load responses were linearized, based on the force corresponding to the physiological range. This allowed us to describe the ligaments as linear and elastic. There were two reasons behind the linearization. Firstly, it reduced the computational complexity of the optimization procedure. Secondly, typical engineering materials exhibit a linear load response in the initial phase of the tensile test.

The upper bound for the material parameters of the mechanism links was obtained by multiplying the highest linearized ligament stiffness—from ALL—by 3. The multiplication was required for mechanisms with custom stiffness responses (see Results). The lower bound for stiffness was arbitrarily set to 20 N/mm. Regarding the geometry of the links, the optimization procedure was allowed to modify each coordinate of the link attachments by ±15 mm.

### 2.4. The Optimization Procedure

Given the equations presented in the previous sections, a mechanism with a defined structure and parameters could be solved in statics and rated on how well it reproduced the angular stiffnesses of the actual IJ. To continually improve the mechanism, by modifying its structure and parameters, we employed sGA. Genetic Algorithms in various implementations have been successfully applied in a variety of problems [40,41,42,43,44,45,46]. In order to explain the working principle of sGA, it is first necessary to introduce Real-Coded Genetic Algorithm (RC-GA)—a global optimization procedure, which works on real-valued decision variable vectors. A basic version of RC-GA takes in a set of randomly generated solutions. It then rates them using the assumed objective function. From the initial set of solutions, a group of parent solutions is selected using a selection procedure. Usually, the better fit solutions (with lower value of the objective) have a higher chance to be included in the parent group. From each two parents in the parent group, a child solution is created using a crossover function. Some initial solutions mutate through a mutation procedure. Finally, the obtained child solutions, mutated solutions, and the best solutions from the initial set are transferred to a new set of solutions and the whole process is repeated. A more detailed explanation of the algorithm, along with different types of selection, crossover, and mutation, is given in [47]. In RC-GA, a solution is always represented by an n-dimensional vector of floating point numbers. These numbers correspond to the parameters of the solution. For instance, in this study, they could represent the geometry of the mechanism. RC-GA is a versatile global optimizer, however, it can only be used for problems in which the solutions have a fixed or predefined structure. Therefore, to employ it for mechanism optimization, the number of links and their type (spring/cable) would have to be set before the optimization. While this approach can return good results, as seen in [30], it is also limiting. Fortunately, extending RC-GA to problems with variable structure can be very simple. In sGA, the structure of the solution is encoded within a single vector of decision variables, along with its parameters. As opposed to RC-GA, this vector actually contains two interdependent sections within it. The first one is composed of n binary values. It controls which ones of the n subsections appearing after it is active in the final solution. This simple division of the vector creates a hierarchical structure within it, which can be applied to optimization of two-platform mechanisms with flexible links. 

As the mechanisms obtained in this study were bound by the ligament system of an actual IJ, they could have a maximum of 9 flexible links (see Figure 1b). Out of them, only 7 were independent, assuming the symmetry in the xy plane. Each link could either be active or inactive and their activity was controlled by the first 7 binary parameters in the decision variable vector. After the structure, the vector contained 7 subsections specifying the geometry, material parameters, and type for each link. The following parameters were contained in every subsection:

- The type (a binary value; 1 corresponded to a spring, while 0 to a cable);
- The stiffness (N/mm);
- The coordinates of the attachment to the lower platform (mm) (2 if the link was in the xy plane or 3 if not);
- The coordinates of the attachment to the upper platform (mm) (2 if the link was in the xy plane or 3 if not);
- The free length (mm).

**Figure 3:**
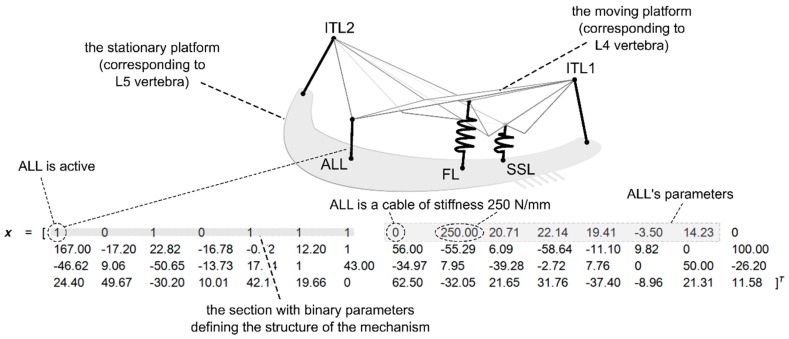
A sample structure with its corresponding decision variable vector in structured Genetic Algorithm (sGA). The image of the platform mechanism based on [30].

An example of the vector with its corresponding mechanism is shown in Figure 3. 

The proposed single-vector representation of the mechanism allowed us to employ sGA for simultaneous optimization of structure and its parameters. The algorithm was implemented using Matlab’s Global Optimization Toolbox as mixed-integer Genetic Algorithm. The settings for it were as follows: The max generations set to 500, the population size set to 60, elite count at 5% of the population size, and the crossover fraction at 82%. The selection function, crossover function, and mutation function remained at their default values (*rank*, *scattered*, and *adaptive_feasible*).

## 3. Results 

### 3.1. The Reference Angular Stiffnesses for the Optimization

The proposed procedure was tested in three different synthesis examples. In the first one, a mechanism with angular stiffnesses based on the actual L4–L5 FSU was obtained. In the second test, the flexion stiffness was significantly increased. For this example, the angular displacement of the mechanism under 10 Nm flexion moment load was to be under 2.00 degrees (deg). This is a significant decrease when compared to an actual IJ, which experiences an angular displacement of about 6.50 degrees under this load. The third case featured a mechanism with significantly reduced ranges of lateral bending and external rotation. The third example featured significantly increased stiffnesses in both lateral bending and external rotation. A typical human IJ experiences an angular displacement of 6.14 degrees and 2.50 degrees under 10 Nm lateral bending and axial rotation under respective moment loads. For this simulation, the displacement was set to 0.50 degrees for both lateral bending and axial rotation under 10 Nm moment loads. The reference angular displacements used in the objective function were specified in Table 1.

### 3.2. Case #1—A Mechanism to Substitute the L4–L5 IJ

**Figure 4:**
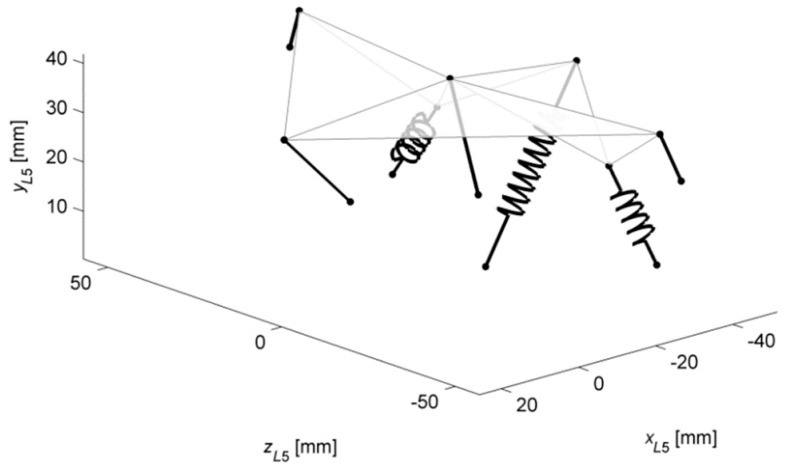
The substitute mechanism for the IJ (Case #1), three-dimensional view.

As mentioned before, in the first application, a mechanism with stiffnesses based on the actual L4–L5 IJ was obtained. The sGA deactivated two links and incorporated three springs in the structure (see Figure 4). This gave the model the ability to transfer moment loads along all three axes of the L5 reference frame with no disc-like structure. 

**Figure 5:**
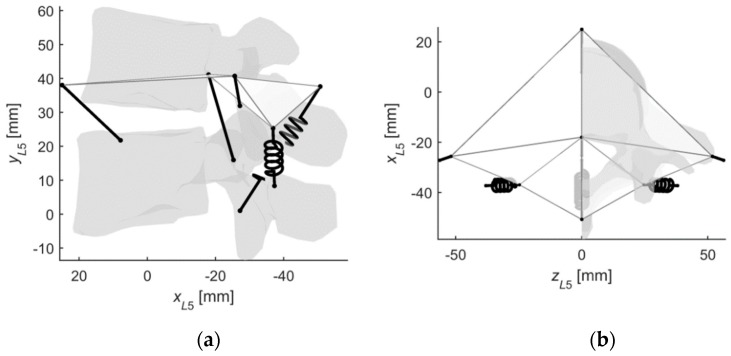
The obtained mechanism with the L4 and the L5 vertebrae for reference: (**a**) Sagittal-plane view; (**b**) Transverse-plane view.

**Figure 6:**
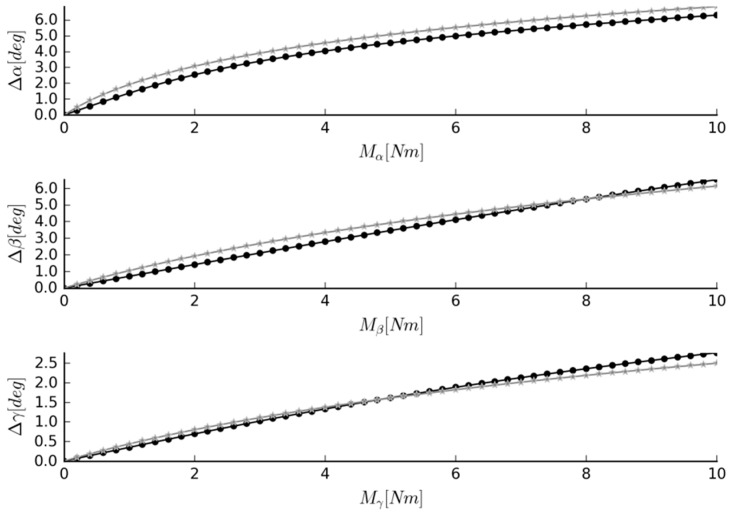
Simulation results for Case #1. Graphs of moment loads acting on the system and angular displacements they cause (black “·” marker = the data obtained from the mechanism, grey “*” marker = the reference data), where α = the flexion, β = the lateral bending angle, and γ = the axial rotation angle.

The procedure also finetuned the material and geometrical parameters of the links. Nevertheless, thanks to the bounds set on the algorithm, the mechanism still resembled the original ligament system, as seen in Figure 4 and Figure 5. The angular displacements of the obtained mechanism were in fair agreement with those of the IJ, as presented in Figure 6. The best results were observed for axial rotation, where the mean difference between the obtained and reference [3] was at 4.21%. The mean differences for flexion and lateral bending were at 7.46% and 5.42%, respectively.

### 3.3. Cases #2 and #3—Mechanisms with Custom Stiffness Profiles

**Figure 7:**
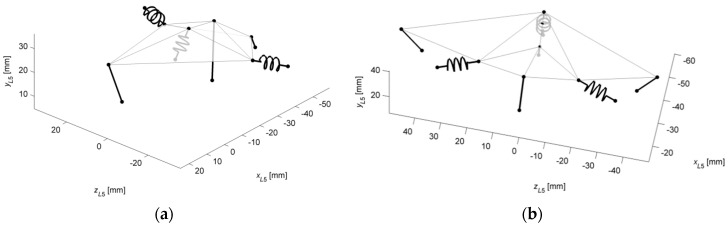
The substitute mechanisms for the IJ in a 3D view: (**a**) Case #2; (**b**) Case #3.

The remaining cases were focused on mechanisms with custom stiffness profiles. In the second case, the target stiffness in flexion was increased, compared to [3], while in the third, the range of lateral bending and the axial rotation were significantly reduced. The obtained mechanisms for both cases are presented in Figure 7. 

The mechanism in Case #2 contained only six links with one symmetrical pair. In total, three of the links were linear springs. In Case #3, the structure was composed of seven elements with two symmetrical pairs of links and three springs.

**Figure 8:**
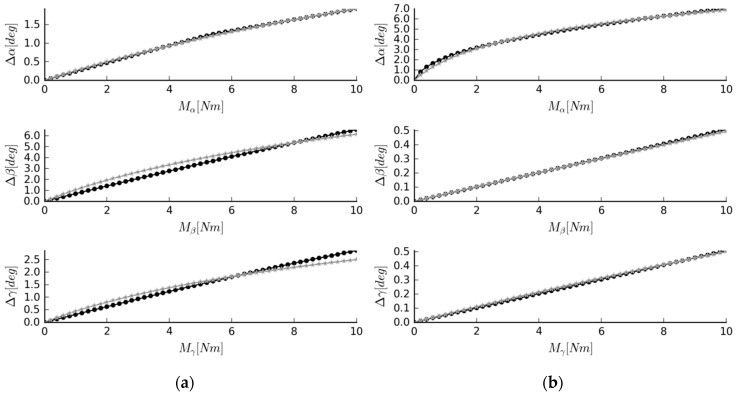
Simulation results for (**a**) Case #2; (**b**) Case #3. Graphs of moment loads acting on the system and angular displacements they cause (black “·” marker—the data obtained from the mechanism, grey “*” marker—the reference data); α—the flexion, β—the lateral bending angle, γ—the axial rotation angle.

In both cases, the obtained angular displacements were in accordance with the target ones (see Figure 8). For Case #2, the best agreement was observed in flexion, with a mean difference of only 0.82%. The mean differences for lateral bending and axial rotation were at 5.71% and 5.74%, respectively. The results obtained for Case #3 were even more promising, with mean differences at 1.45%, 0.74%, and 1.42 for flexion, lateral bending, and axial rotation, respectively.

## 4. Discussion

In this study, an automated method for designing substitute mechanisms for a human intervertebral joint was proposed. The mechanisms were obtained through optimization with structured Genetic Algorithm, with the objective based on the reference angular displacements under specified moment loads. The algorithm simultaneously optimized the structural and material parameters of mechanisms with two rigid platforms, connected by flexible links. The approach was tested in three numerical scenarios. In the first test, a mechanism with angular stiffnesses corresponding to the actual L4–L5 intervertebral joint was obtained. Scenarios 2 and 3 featured mechanisms with geometry and structure comparable to the joint, but with custom stiffness profiles. In all cases, the obtained results proved the effectiveness of the method. 

As mentioned in the introduction, the proposed approach was an extension of a previously published one [30], where the structure optimization and the parameter estimation were two independent procedures. When comparing the IJ substitute obtained in [30] to the current one (Case #1), it is notable that both had seven links with three springs. Furthermore, the quality of their responses to loads was comparable. Nevertheless, some interesting differences in their structures could be observed. The current one contained two pairs of symmetrical links and only one spring in the xy plane, as opposed to one symmetrical pair and three springs in the xy plane of the substitute from [30]. 

The IJ substitute obtained with our approach (Case #1) distinguished itself from the currently available substitutes [16,28,29], with its angular stiffnesses closely mimicking the IJ. In comparison to the actual IJ and its models [3,8,9,10], the mechanism retained a simple structure of only seven flexible links. Despite having no elements corresponding to the disc and the facets, it offered the desired load responses in three dimensions. As mentioned in the introduction, typical spine-segment substitutes feature some form of a central element—a spring or a beam, which mostly corresponds to the disc. In this case, the behavior of the disc was incorporated into the springs in the system. While this has been done previously [30], in this study, the structure and the parameters were optimized simultaneously. This allowed us to obtain structures based on the IJ, but with drastically different stiffness responses.

In some cases, the overall similarity to the IJ may be necessary, but with some parts of the static behavior altered for a specific task. To test if the proposed procedure could be employed in cases where some aspects of the static behavior were drastically different from the IJ, we performed two extra simulations (Cases #2 and #3). Case #2 featured a significantly increased angular stiffness in flexion, while Case #3 had a reduced range of motion in lateral bending and axial rotation. In both of them, the optimization procedure returned structures resembling the IJ, with the mean difference between the custom and the obtained displacements under the same moment loads never exceeding 6%. It is also worth mentioning that the procedure reduced the number of links in the system in all the considered cases. The bounds for the mechanisms were generated based on the ligament system of the IJ, composed of nine linear cables. The optimized mechanisms contained between 6 and 7 links. This suggests some possible future extensions of the method—the number of links could be included in the objective function so that the procedure would favor simpler structures. 

Regarding the choice of the optimization algorithm, we initially considered different optimization methods. Our first choice was the local methods, such as Nelder-Mead. Nevertheless, due to very limited success, we quickly gave up on them. The mechanisms obtained with these methods were often unable to transfer all of the assumed loading conditions. In the area of global optimization, we considered some alternatives to sGA. Our first idea was to utilize RC-GA with vectors of variable length. Nevertheless, we were discouraged by the difficulties in implementing genetic operators for it—mainly crossover. This was also pointed out by other research groups [48]. Our next approach was to employ Genetic Programming. In Genetic Programming, the solution is represented by a tree rather than a vector. All of the genetic operators are made to work on the tree and its branches. Nevertheless, in this study, the structure only has one level—the links connect two platforms, one of which is stationary. Representing these structures with trees would needlessly complicate the implementation and slow down the computation, especially in high-level languages. We believe that this approach would be very promising, but for multi-level structures. On the other hand, sGA does not require new definitions for crossover, as with variable-vectors RC-GA, and the structure is fully described with one vector, as opposed to a more complex tree in genetic programming. Furthermore, it is also very simple to implement sGA in high-level languages, such as Matlab, which makes the study easier to reproduce and reuse.

The limitations of the approach in its current form are mainly in the considered objectives. While the obtained mechanisms reproduce the target angular stiffnesses, it is also necessary to considered other parts of their behavior. In this regard, the two major objectives to include would be: An objective that rates the stress-strain state in the mechanism and an objective that considers the interelement collisions in the mechanism. These two objectives are different in nature to the considered ones. Therefore, it would also be advisable to consider more advanced formulations for multi-objective optimization, using a Pareto concept or a Pareto concept combined with a weighted summation strategy [49,50].

Considering the results obtained for the IJ substitute (Case #1) and the custom mechanisms (Cases #2 and #3), the proposed approach was proven to be effective. It could be employed in the design of artificial joints for humanoid robots and orthotic structures for the human spine. As the approach is general, it could also be transferred to different body joints.

## Figures and Tables

**Table 1:** The target angular displacements under the moment loads (“as in reference [3]” refers to the displacements based on an actual verified model of the IJ presented in [3]).

Case Number	Δα [deg]	Δβ [deg]	Δγ [deg]
Case #1	as in Reference [3]	as in Reference [3]	as in Reference [3]
Case #2	log(Mα5.40+1)0.34	as in Reference [3]	as in Reference [3]
Case #3	as in Reference [3]	log(Mβ101.70+1)0.19	log(Mγ36.11+1)0.49
